# Walking cadence (steps/min) and intensity in 61–85-year-old adults: the CADENCE-Adults study

**DOI:** 10.1186/s12966-021-01199-4

**Published:** 2021-09-23

**Authors:** Catrine Tudor-Locke, Jose Mora-Gonzalez, Scott W. Ducharme, Elroy J. Aguiar, John M. Schuna, Tiago V. Barreira, Christopher C. Moore, Colleen J. Chase, Zachary R. Gould, Marcos A. Amalbert-Birriel, Stuart R. Chipkin, John Staudenmayer

**Affiliations:** 1 College of Health and Human Services, University of North Carolina at Charlotte, 9201 University City Blvd., Charlotte, NC USA; 2 Department of Kinesiology, California State University, Long Beach, Long Beach, CA USA; 3 Department of Kinesiology, The University of Alabama, Tuscaloosa, AL USA; 4 School of Biological and Population Health Sciences, Oregon State University, Corvallis, OR USA; 5 Exercise Science Department, Syracuse University, Syracuse, NY USA; 6 Department of Epidemiology, University of North Carolina at Chapel Hill, Chapel Hill, NC USA; 7 Department of Kinesiology, University of Massachusetts Amherst, Amherst, MA USA; 8 Department of Mathematics and Statistics, University of Massachusetts Amherst, Amherst, MA USA

**Keywords:** Accelerometer, Exercise, Pedometer, Physical activity, Step rate

## Abstract

**Background:**

Heuristic (i.e., evidence-based, rounded) cadences of ≥100 and ≥ 130 steps/min have consistently corresponded with absolutely-defined moderate (3 metabolic equivalents [METs]) and vigorous (6 METs) physical activity intensity, respectively, in adults 21–60 years of age. There is no consensus regarding similar thresholds in older adults.

**Purpose:**

To provide heuristic cadence thresholds for 3, 4, 5, and 6 METs in 61–85-year-old adults.

**Methods:**

Ninety-eight community-dwelling ambulatory and ostensibly healthy older adults (age = 72.6 ± 6.9 years; 49% women) walked on a treadmill for a series of 5-min bouts (beginning at 0.5 mph with 0.5 mph increments) in this laboratory-based cross-sectional study until: 1) transitioning to running, 2) reaching ≥75% of their age-predicted maximum heart rate, or 3) reporting a Borg rating of perceived exertion > 13. Cadence was directly observed and hand-tallied. Intensity (oxygen uptake [VO_2_] mL/kg/min) was assessed with indirect calorimetry and converted to METs (1 MET = 3.5 mL/kg/min). Cadence thresholds were identified via segmented mixed effects model regression and using Receiver Operating Characteristic (ROC) curves. Final heuristic cadence thresholds represented an analytical compromise based on classification accuracy (sensitivity, specificity, positive and negative predictive value, and overall accuracy).

**Results:**

Cadences of 103.1 (95% Prediction Interval: 70.0–114.2), 116.4 (105.3–127.4), 129.6 (118.6–140.7), and 142.9 steps/min (131.8–148.4) were identified for 3, 4, 5, and 6 METs, respectively, based on the segmented regression. Comparable values based on ROC analysis were 100.3 (95% Confidence Intervals: 95.7–103.1), 111.5 (106.1–112.9), 116.0 (112.4–120.2), and 128.6 steps/min (128.3–136.4). Heuristic cadence thresholds of 100, 110, and 120 were associated with 3, 4, and 5 METs. Data to inform a threshold for ≥6 METs was limited, as only 6/98 (6.0%) participants achieved this intensity.

**Conclusions:**

Consistent with previous data collected from 21–40 and 41–60-year-old adults, heuristic cadence thresholds of 100, 110, and 120 steps/min were associated with 3, 4, and 5 METs, respectively, in 61–85-year-old adults. Most older adults tested did not achieve the intensity of ≥6 METs; therefore, our data do not support establishing thresholds corresponding with this intensity level.

**Trial registration:**

Clinicaltrials.gov NCT02650258. Registered 24 December 2015.

**Supplementary Information:**

The online version contains supplementary material available at 10.1186/s12966-021-01199-4.

## Introduction

The application and interpretation of step-based metrics (e.g., steps/day) in physical activity assessment and intervention is now widely accepted [1, 2]. The rise in step-based metrics is burgeoned in large part by the collective and widespread commercial enterprise that has produced an abundant and affordable variety of wearable technologies with step-counting features, including cadence (i.e., steps/min) [3]. As opposed to measuring a volume of activity (steps/day), cadence is a rate representing quantified steps displayed over time. Based largely on studies of level or near level walking, cadence is strongly (r = 0.94) [4] and consistently [5–12] associated with physical activity intensity. The strength of the relationship is such that a cadence of ≥100 steps/min is now clearly established as a heuristic (i.e., rounded, generalized, yet evidence-based) threshold indicative of absolutely-defined moderate intensity for level or near level walking expressed in terms of mass-specific oxygen cost (i.e., 3 metabolic equivalents [METs]; 1 MET = 3.5 mL/kg/min), at least for younger and middle-aged adults [5–10]. In previous reports arising from the CADENCE-Adults study [11, 13], we again confirmed this finding in 21–40-year-old [11] and 41–60-year-old [13] adults. We also demonstrated that ≥130 steps/min was a consistent indicator of absolutely-defined vigorous intensity (i.e., ≥ 6 METs) in both age groups [11, 13].

The most physically inactive age group worldwide is comprised of adults ≥60 years of age, known to spend approximately 80% of their time in either light physical activity or sedentary behavior [14, 15]. Accessible and easy-to- understand physical activity metrics, including intensity indices, are needed that also apply to older adults. Although cadence thresholds have been successfully identified for 21–40-year-old [11] and 41–60-year-old [13] adults, it is not clear whether the same heuristic thresholds proposed for young and middle-age adults can also be used in ambulatory and ostensibly healthy older adults. A recent meta-analysis [16] showed that the gross and net metabolic cost of walking (assessed as speed, not cadence) is approximately 12 and 17%, respectively, higher in older adults compared to younger adults, however, the authors concluded that it was unclear from the literature reviewed whether the apparent difference was directly caused by age, by an interaction between age and methodology, or by the walking strategy. Concerning the later possibility, cautious gait, in which older people take shorter steps to increase stability, increases co-activation of hip, knee, and ankle muscles to increase stability and also reduces passive leg swing dynamics, further increasing muscular, and likely the metabolic, cost of walking.

To date, there are only two studies [12, 17] to propose cadence thresholds based on absolutely-defined intensity in older adults (ranging in these studies from 60–87 years of age). Although both studies assessed cadence via direct observation [12, 17], they used limited sample sizes (19–29 participants) and study designs differed in various ways. Specifically, these studies used different treadmill testing protocols (i.e., self-selected [17] vs. pre-established walking speeds [12]), and analyzed their data using distinctly different statistical approaches (i.e., linear [17] vs. curvilineal [12] model forms), which may explain why they found cadence estimates associated with absolute moderate intensity that ranged from 99 steps/min [17] to 104–108 steps/min [12]. Thus, additional research is needed to address inconsistencies and knowledge gaps using a more robust study design (i.e., larger and balanced sample size, standardized protocols, objective measurements, rigorous statistical analyses, etc.).

The overall aim of the CADENCE-Adults study was to identify heuristic cadence thresholds associated with metabolic intensity during treadmill walking in adults across the full adult lifespan (21–85 years of age) [11, 13]. The specific aims of this installment of the CADENCE-Adults study were to: 1) characterize the relationship between cadence and absolutely-defined intensity, including evaluating potential modifiers (e.g., sex, age, leg length, or body mass index [BMI]) of the relationship, and 2) identify heuristic cadence thresholds indicative of 3, 4, 5, and 6 METs in ambulatory and ostensibly healthy older adults 61–85 years of age.

## Methods

### Study design and regulatory information

As registered on ClinicalTrials.gov (NCT02295072) and as previously reported [11, 13], the CADENCE-Adults study was a laboratory-based cross-sectional study of cadence and intensity in 21–85-year-old adults. The University of Massachusetts Amherst Institutional Review Board approved the study protocol. The specific data reported herein, focused on 61–85-year-old adults, were collected between November 2018 and August 2019 in the Physical Activity and Health Laboratory at the University of Massachusetts Amherst.

### Participants

The study was designed to enroll a sex-and-age balanced sample of 100 ambulatory individuals, comprised of 10 men and 10 women for each 5-year age category between 61–85 years (61–65, 66–70, 71–75, 76–80, 81–85 years of age). This strategy of recruitment favored minimization of bias sources and generalizability of the findings. We used word-of mouth, newspaper and radio advertisements, electronic postings, e-mails, posted flyers, and general recruitment events (i.e., community centers, retirement villages and assisted living centers) as recruitment strategies. Once we were contacted by a potential participant, we first carried out a phone screening and identified eligible participants and scheduled an in-person confirmatory screening process leading up to obtaining written informed consent prior to data collection procedures. Individuals who used wheelchairs or walking aids, or had other impairments to normal ambulation were excluded due to the intended focus of the study on ambulatory activity. Participants were also excluded if they presented/reported any of the following conditions: Stage 2 hypertension (i.e., systolic blood pressure ≥ 160 mmHg or diastolic blood pressure ≥ 100 mmHg), hospitalized for mental illness in the last 5 years, smoking, a BMI < 18.5 kg/m^2^ or > 40 kg/m^2^, any condition or medication that could affect heart rate response to exercise, a stroke or any cardiovascular disease, pacemakers or other implants, or being pregnant. All participants were approved by our medical investigator (SRC) for exercise following a resting electrocardiogram.

### Measurements and procedures

Following a minimal 4 h fast and prior to arrival at the testing site, participants’ standing height, weight, leg length, waist circumference, BMI, and self-classified race/ethnicity were assessed as described in detail previously [11].

The treadmill testing protocol has also been reported previously [11, 13] but is repeated again here for clarity. Participants wore a Jaeger Oxycon Mobile (CareFusion BD Germany 234 GmbH, Höchberg, Germany) portable indirect calorimeter to measure oxygen consumption (VO_2_) in mL/kg/min. Heart rate was tracked using a Polar T31 Coded Transmitter (Polar Kempele, Finland) chest strap. Once seated VO_2_ values were established for at least 5 min, participants then walked on the treadmill for up to twelve 5-min bouts (with a 2-min standing rest between bouts) at a 0% grade and speeds ranging in 0.5 mph increments from 0.5 mph (0.8 km/h) up to 6.0 mph (9.7 km/h). During the last minute of each treadmill bout participants were asked to report their rating of perceived exertion (RPE) using the Borg scale [18]. Steps were directly observed and recorded using a hand-tally counter during each treadmill bout, and also recorded with video camera. Consistent with the previous CADENCE-Adults reports [11, 13], treadmill testing was terminated at the end of the 5-min bout when the participant: 1) naturally transitioned from walking to running; 2) achieved ≥75% of age predicted heart rate maximum [0.75 x (220 - age)] [19]; and/or 3) reported an RPE > 13 [19]. Additionally, either the research staff or the participant could terminate the protocol for any reason, including, for example, perceived fatigue or safety concerns.

### Analytic sample

After screening and enrollment, two women (84.5 ± 0.7 years of age) did not initiate the treadmill protocol due to safety concerns identified as unsteadiness during normal ambulation. The remaining 98 participants (72.6 ± 6.9 years of age; 49% women) produced data for 567 treadmill bouts. Reasons for testing termination of these 98 participants were: 1) exceeding 75% of age predicted heart rate maximum (*n* = 69); 2) self-reporting an RPE > 13 (*n* = 22), 3) voluntarily deciding to stop (n = 2); or 4) researcher decision to terminate the test due to safety concerns (n = 2). Additionally, 3 participants ran during their final bout and, in order to maintain the analytical focus herein on walking data, these three specific running bouts were excluded from analysis (i.e., all their walking bouts remained in the data set). Thus, the final analytical data set (Additional file 1 and corresponding data dictionary, Additional file 2) comprises 98 participants and 564 treadmill walking bouts.

### Data management and statistical analyses

Steps (cadence) and VO_2_ data were managed using MATLAB (The MathWorks, Natick, MA) as previously reported [11, 13]. Statistical analyses were performed using R-Studio (version 3.6.2, R Foundation for Statistical Computing, Vienna, Austria). Continuous variables were calculated as means and standard deviations and categorical variables as frequencies. Statistical significance was interpreted using α set at 0.05.

Based on what we learned from the original study [11], a segmented regression model with fixed and random coefficients was used to analyze the cadence-intensity relationship as two distinct linear portions (i.e., different slopes and intercepts) separated by an identifiable segmented break point value that minimized mean square error. Marginal R^2^ values, as well as the slopes and 95% confidence intervals (CIs) for fixed effects, were reported as a description of model fit. Considering that previous research in older adults reported a potential modifying effect of age and height [17], or BMI [12] on the relationship between cadence and absolutely-defined intensity in older adults, we performed separate segmented regression models including sex, age, leg length (which has shown a strong correlation with height [7]), or BMI as additional factors to evaluate their effect. Again, and consistent with our earlier reports [11, 13], marginal R^2^ values were reported to describe whether models that included sex, age, leg length, or BMI improved overall model fit.

The threshold for absolutely-defined moderate intensity was identified at ≥3 METs [20, 21], with additional incremental values of 4 and 5 METs. The threshold for vigorous intensity was identified at ≥6.0 METs [20]. Specifically, incremental cadence thresholds corresponding to ≥3, 4, 5 and 6 METs were calculated using the model’s regression equation and ± 95% prediction intervals (PIs). Sensitivity, specificity, positive predictive value, negative predictive value (NPV), and overall accuracy were defined and calculated consistent with our previous reports [11, 13] to assess classification accuracy of each regression-identified threshold. ROC curve analysis using Youden’s index [22] was also performed to identify optimal cadence thresholds related to ≥3, 4, 5 and 6 METs. Classification accuracy analyses were again performed to obtain sensitivity, specificity, PPV, NPV, overall accuracy, and area under the curve (AUC; bootstrapping using 20,000 replicates) of these ROC-identified cadence thresholds. Also consistent with our previous reports [11, 13], AUC values were interpreted as excellent (≥ 0.90), good (0.80–0.89), fair (0.70–0.79), and poor (< 0.70) [23].

Following the approach described in the original report of the CADENCE-Adults study [11], heuristic cadence thresholds were established as rounded multiples of 5 steps/min considering the more precise MET-related estimates generated from the segmented regression model and ROC curves. In cases where the two analytical approaches produced discrepant estimates, the trade-offs in sensitivity, specificity, PPV, NPV, and overall accuracy were systematically considered for each candidate threshold prior to ultimately settling on a single heuristic threshold corresponding to ≥3, 4, 5, and 6 METs. As previously reported [11, 13], our ultimate decisions were guided by an intention to create a harmonious and incremental set of cadences that tolerated false negatives over false positives and thus was most useful from a public health perspective. Finally, we determined classification accuracy (based again on sensitivity, specificity, PPV, NPV and overall accuracy) of the candidate heuristic thresholds for identifying incremental levels of MET-defined intensity.

## Results

### Descriptive characteristics

Table 1 presents the descriptive characteristics of the analytic sample. By design, the sample was evenly distributed by sex (*n* = 50 men; *n* = 48 women) and age (men’s age = 72.8 ± 7.0 years; women’s age = 72.4 ± 6.8 years) as per our strategic recruitment plan. Descriptive data including sample sizes, cadences, VO_2_, and MET values for each treadmill bout are presented in Table 2. Only 3 male participants (62.7 ± 2.1 years of age) reached the maximum reported speed of 4.5 mph and only 6 participants (64.5 ± 3.6 years of age; 1 woman) reached a vigorous intensity of ≥6 METs.

**Table 1:** Descriptive characteristics of the participants

Variable	Men (*n* = 50)		Women (*n* = 48)		Total (*n* = 98)	
	Mean	SD	Mean	SD	Mean	SD
Age (years)	72.8	7.0	72.4	6.8	72.6	6.9
Weight (kg)	79.9	9.5	65.1	11.0	72.7	12.6
Height (cm)	173.1	6.4	161.2	5.7	167.3	8.5
Leg length (cm)	83.0	4.4	76.7	3.9	79.9	5.2
BMI (kg/m^2^)	26.7	2.7	25.1	4.0	25.8	3.5
	n	%	n	%	n	%
BMI classifications						
Normal weight	17	34.0	26	54.2	43	43.9
Overweight	29	58.0	16	33.3	45	45.9
Obese	4	8.0	6	12.5	10	10.2
Race						
White	42	84.0	42	87.5	84	85.7
American Indian/Alaska Native	2	4.0	0	0.0	2	2.0
Asian	0	0.0	1	2.1	1	1.0
Native Hawaiian/Pacific Islanders	0	0.0	0	0.0	0	0.0
African-American	1	2.0	0	0.0	1	1.0
More than one race	0	0.0	1	2.1	1	1.0
Unknown or not reported	5	10.0	4	8.3	9	9.2
Ethnicity						
Not Hispanic or Latino	42	84.0	43	89.6	85	86.7
Hispanic or Latino	0	0.0	0	0.0	0	0.0
Unknown or not reported	8	16.0	5	10.4	13	13.3

BMI = Body Mass Index. BMI categories: normal or healthy weight (18.5–24.9 kg/m^2^), overweight (25.0–29.9 kg/m^2^), obese (≥ 30 kg/m^2^) [36]

**Table 2:** Sample sizes, cadences, VO_2_, and METs for treadmill bouts

Treadmill Speed (mph)	***n***	Cadence (steps/min)	Min–Max	VO_**2**_ (mL/kg/min)	Min–Max	METs	Min–Max
0.5	98	69.3 ± 18.8	38–131	7.6 ± 1.3	4.2–12.8	2.2 ± 0.4	1.2–3.7
1.0	89	80.6 ± 14.0	51–120	8.4 ± 1.2	4.9–11.9	2.4 ± 0.4	1.4–3.4
1.5	86	90.6 ± 10.8	66–125	9.1 ± 1.3	6.1–13.1	2.6 ± 0.4	1.8–3.7
2.0	81	98.6 ± 8.4	81–130	10.0 ± 1.3	6.5–14.2	2.8 ± 0.4	1.9–4.1
2.5	75	106.3 ± 7.0	93–128	11.1 ± 1.5	7.3–15.6	3.2 ± 0.4	2.1–4.5
3.0	67	114.1 ± 6.5	101–132	13.2 ± 1.5	9.5–17.0	3.8 ± 0.4	2.7–4.8
3.5	48	121.2 ± 7.9	106–144	16.2 ± 1.9	12.7–20.2	4.6 ± 0.5	3.6–5.8
4.0	17	129.4 ± 8.3	113–148	19.2 ± 2.4	15.5–24.7	5.5 ± 0.7	4.4–7.1
4.5	3	136.9 ± 7.9	129–145	24.1 ± 2.2	22.7–26.6	6.9 ± 0.6	6.5–7.6

### Segmented regression with random coefficients model

**Fig. 1:**
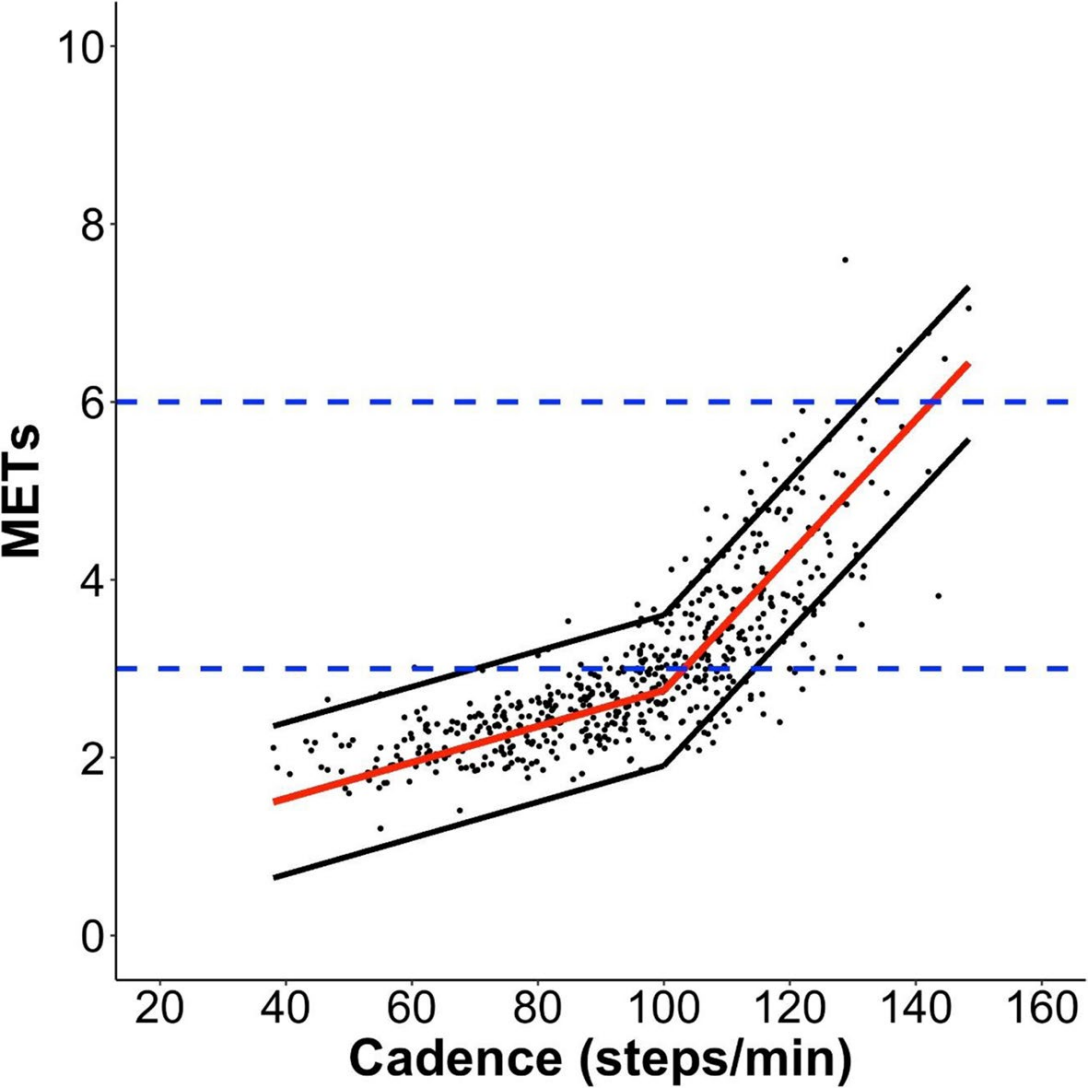
Relationship between cadence and METs using a segmented regression model with random coefficients in older adults. Breakpoint is at 100 steps/min; marginal R^2^ = 0.74. Red line represents the mean MET values (y–axis) for each corresponding cadence value (x–axis), and the black lines represent the 95% Prediction Intervals. Blue horizontal dotted lines indicate moderate (3 METs) and vigorous intensities (6 METs)

The segmented regression model of the cadence-intensity relationship displayed two separate linear regions and produced a best fit using a breakpoint at 100 steps/min (marginal R^2^ = 0.74) (Fig. 1). The model also yielded a pre-breakpoint slope of 0.020 (95% CI: 0.018–0.023), and a post-breakpoint slope of 0.076 (95% CI: 0.072–0.080), and the following equations: 1) if cadence ≤100 steps/min or intensity ≤2.8, METs = 0.731 + 0.020 * cadence; and alternatively, 2) if cadence > 100 steps/min or intensity > 2.8 METs, METs = − 4.850 + 0.076 * cadence. Analyses accounting for the potential modifying effect of sex, age, leg length, or BMI on the cadence-intensity relationship revealed that adding sex or leg length to separate models did not result in a different breakpoint from the original segmented regression model. Adding age or BMI slightly changed the originally-identified 100 steps/min breakpoint to 99.2 and 99.4 steps/min, respectively. Including sex, age, leg length, or BMI as individual predictors considered together with cadence did not improve the predictive capabilities of the original segmented regression model. In all cases a marginal R^2^ = 0.74 was observed. Table 3 reports cadence thresholds estimated using the regression model: ≥ 103.1 (95% PI: 70.0–114.2), 116.4 (105.3–127.4), 129.6 (118.6–140.7), and 142.9 steps/min (131.8–148.4) corresponding to ≥3, 4, 5, and 6 METs intensities, respectively.

**Table 3:** Cadence thresholds (steps/min) for absolutely-defined moderate and vigorous intensity based on regression and ROC curve analyses

Intensity METs	Measure	Regression thresholds		ROC thresholds		Heuristic thresholds
		Value	95% PI	Value	95% CI	Value
3	Threshold (steps/min)	**103.1**	70.0–114.2	**100.3**	95.7–103.1	**100**
	Se	81.6	–	86.8	–	86.8
	Sp	86.6	–	84.5	–	83.3
	PPV	80.5	–	79.2	–	78.0
	NPV	87.4	–	90.4	–	90.3
	Accuracy	84.6	–	85.5	–	84.8
	AUC	**–**	–	0.93	0.91–0.95	**–**
4	Threshold (steps/min)	**116.4**	105.3–127.4	**111.5**	106.1–112.9	**110**
	Se	68.2	–	88.2	–	89.4
	Sp	93.5	–	87.3	–	84.1
	PPV	65.2	–	55.1	–	50.0
	NPV	94.3	–	97.7	–	97.8
	Accuracy	89.7	–	87.4	–	84.9
	AUC	**–**	–	0.94	0.92–0.96	**–**
5	Threshold (steps/min)	**129.6**	118.6–140.7	**116.0**	112.4–120.2	**120**
	Se	44.4	–	96.3	–	81.5
	Sp	98.5	–	87.9	–	91.6
	PPV	60.0	–	28.6	–	32.8
	NPV	97.2	–	99.8	–	99.0
	Accuracy	95.9	–	88.3	–	91.1
	AUC	**–**	–	0.96	0.94–0.98	**–**
6	Threshold (steps/min)	**142.9**	131.8–148.4	**128.6**	128.3–136.4	**130**
	Se	33.3	–	100.0	–	83.3
	Sp	99.8	–	97.1	–	97.5
	PPV	66.7	–	27.3	–	26.3
	NPV	99.3	–	100.0	–	99.8
	Accuracy	99.1	–	97.2	–	97.3
	AUC	**–**	–	0.99	0.98–1.00	–

Segmented regression and Receiver Operating Characteristic (ROC) thresholds are represented as means (95% Prediction Intervals) for segmented regression and means (99% Confidence Intervals) for ROC. Trade-offs in terms of Sensitivity (Se), Specificity (Sp), Positive Predictive Value (PPV), Negative Predictive Value (NPV) and overall accuracy between the thresholds derived from the segmented regression and ROC analyses were considered to select heuristic thresholds. Selected heuristic thresholds reflect a purposely favored tolerance for false-negative versus false-positive classifications. AUC = Area under the curve, CI = Confidence Intervals, PI = Prediction Intervals

### Receiver operating characteristic (ROC) analysis

Table 3 also presents cadence thresholds for incremental levels of intensity identified using the ROC analysis. ROC-specific cadence-intensity thresholds corresponding to ≥3, 4, 5, and 6 METs were ≥ 100.3 (95% CI: 95.7–103.1), 111.5 (106.1–112.9), 116.0 (112.4–120.2), and 128.6 (128.3–136.4) steps/min, respectively. Sensitivity and specificity values for all cadence intensity thresholds were > 85% and overall accuracy values were between 85 and 97%. Also, AUC values were ≥ 0.93 for all thresholds, indicative of an excellent classification accuracy.

### Heuristic thresholds

Table 3 presents heuristic cadence thresholds (rounded to the nearest 5 steps/min) related to increments of METs-defined intensity that were selected based on congruence between the regression and ROC analysis and after considering the trade-offs in sensitivity, specificity, PPV, NPV, and overall accuracy. Heuristic cadence thresholds of ≥100, 110, and 120 steps/min emerged for ≥3, 4, and 5 METs. The sensitivity values associated with these thresholds were ≥ 81% and the specificity values were all ≥83%. For ≥6 METs, we compared the classification accuracy indices between ≥125 and 130 steps/min (see Additional file 3 and Table 3, respectively). Using a heuristic threshold of ≥125 steps/min to classify walking at ≥6 METs resulted in a sensitivity = 100%, a specificity = 94.4%, a PPV = 16%, an NPV = 100%, and an overall accuracy = 95%, while using ≥130 steps/min resulted in sensitivity = 83.3%, a specificity = 97.5%, a PPV = 26%, an NPV = 100%, and an overall accuracy = 97%.

**Fig. 2:**
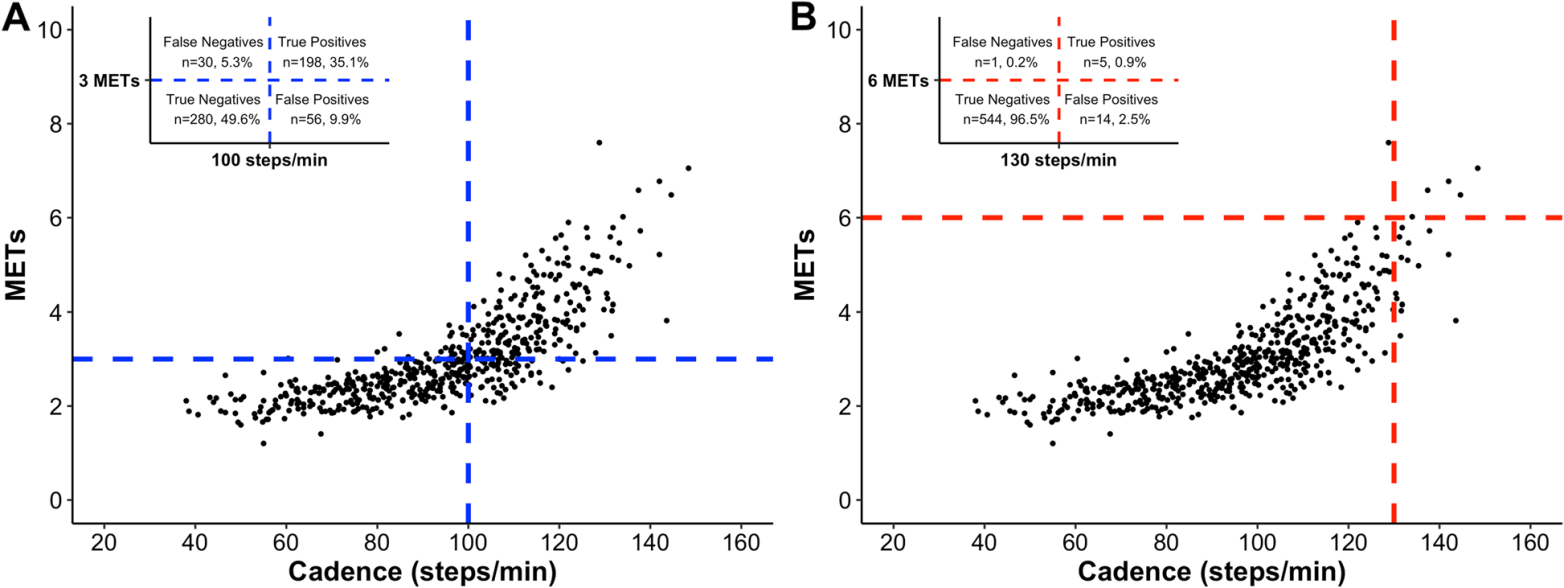
Classification accuracy of heuristic cadence thresholds and MET intensities. A) ≥ 100 steps/min and ≥ 3 METs, B) ≥ 130 steps/min and ≥ 6 METs)

Figure 2 reports the classification accuracy indices for heuristic cadence thresholds of ≥100 and ≥ 130 steps/min relative to their associated MET-defined intensities. When the ≥100 steps/min heuristic cadence threshold was applied for ≥3 METs, 85% of all bouts (i.e., 478 bouts of the total 564) were correctly classified (i.e., overall accuracy: true positives plus true negatives). When using the heuristic cadence threshold of ≥130 steps/min, 97% of all bouts (i.e., 549 bouts of the total 564) were correctly classified, although only five bouts (1%) were classified as true positives and the rest of the rest 544 bouts (96%) as true negatives. For comparison purposes, an Additional file 4 graphically represents the classification accuracy of ≥125 steps/min as a candidate heuristic cadence threshold associated with ≥6 METs. When using ≥125 steps/min as heuristic threshold, 95% of all bouts (i.e., 533 bouts of the total 564) were correctly classified, and in this case the 6 bouts (1.1%) ≥ 6 METs were classified as true positives. As we pointed out previously, only 6 participants (64.5 ± 3.6 years of age; 1 woman) reached a vigorous intensity of ≥6 METs (5 bouts classified as true positives plus 1 bout classified as false negative).

## Discussion

This analysis focused on data collected from ambulatory and ostensibly healthy older adults (61–85 years of age) during the larger sex- and age-balanced CADENCE-Adults study of cadence and absolutely-defined intensity during treadmill walking. It builds upon previous findings based on 21–40-year-old [11] and 41–60-year-old adults [13]. The results herein confirm that ≥100 steps/min is a heuristic cadence threshold associated with moderate intensity (i.e., ≥ 3 METs) in older adults. Considering these findings along with our previous reports on younger and middle-aged adults [11, 13], 100 steps/min appears to be a valid heuristic threshold for absolutely-defined moderate intensity across the full adult lifespan of ambulatory and ostensibly health adults (i.e., encompassing 21–85 years of age). These data are also consistent with ≥130 steps/min being a heuristic cadence threshold associated with absolutely-defined vigorous intensity (i.e., ≥ 6 METs), in alignment with the results we previously reported in the two younger age groups [11, 13]. However, the small sample of older adults (*n* = 6) achieving ≥6 METs is inadequate for confident calibration of this threshold. Further investigation is needed to evaluate whether walking at ≥6 METs is practically achievable for older adults and, if so, verify the corresponding cadence threshold. Finally, heuristic cadence thresholds of ≥110 and 120 steps/min were associated with ≥4 and 5 METs, respectively, across the adult lifespan.

We first reported the potential for establishing cadence thresholds for classifying walking intensity, and specifically ≥100 steps/min associated with ≥3 METs, in 2005 with a small younger adult sample [6]. Subsequently, a growing number of studies conducted with predominantly younger and middle-aged adults (i.e., < 60 years of age) [5–13] consistently confirmed this threshold to achieve an absolutely-defined moderate intensity. The very few studies that have previously been conducted with older adult samples [12, 17, 24] have reported inconsistent results, hampering our ability to provide clear cadence recommendations for this age group. Applying consistent definitions, protocols, and analytical strategies, herein we confirmed ≥100 steps/min as a heuristic cadence threshold associated with absolutely-defined moderate intensity with an excellent classification (as evidenced by AUC = 0.93 derived from the ROC analysis) and an overall accuracy of 85% for classifying true positives and true negatives. These findings are consistent with the two previous CADENCE-Adults installments in 21–40-year-old [11] and 41–60-year-old adults [13] in which we also observed an excellent classification (as evidenced by AUC = 0.95 and 0.97, respectively) and an overall accuracy of 87 and 89%, respectively.

We must acknowledge that the finding of ≥100 steps/min as a heuristic cadence threshold associated with an absolutely-defined moderate intensity of ≥3 METs for the full adult lifespan (21–85 year) [11, 13] may appear contradictory in comparison to what was declared by a recent meta-analysis [16]. In particular, this meta-analysis indicated that older adults experience a higher metabolic cost of walking at similar speeds compared with younger adults [16]. It is important to clarify that, although cadence is one of the temporal factors underlying speed (along with step length), it is not necessarily an interchangeable metric with speed. For example, older adults may increase cadence to protect against declines in walking speed relative to younger adults [25]. Increasing cadence at similar walking speeds may be an additional explanation of why metabolic costs differ across the adult lifespan [26]. Herein, we focus on cadence as a metric of intensity, not speed. The question regarding potential age-related differences in metabolic cost of walking at similar speeds is not within the specific scope of this research project. Regardless, combining our findings from the two previous CADENCE-Adults reports [11, 13] and this current one now clearly demonstrates that the relationship between cadence and intensity is such that we can confidently interpret ≥100 steps/min as indicative of absolutely-defined moderate intensity ambulatory activity across the adult lifespan.

As indicated above, the results regarding absolutely-defined vigorous intensity were not as clear and consistent. The heuristic cadence of ≥130 steps/min showed a better classification of ≥6 METs than the alternative candidate value of ≥125 steps/min, as evidenced by a higher probability (i.e., PPV) of 26% versus 16% of achieving that vigorous intensity threshold. Also, ≥ 130 steps/min showed a higher accuracy than ≥125 step/min, with 97% versus 94% of bouts being correctly classified, respectively. These findings are in general agreement with those of the CADENCE-Adults report focused on 41–60-year-old adults [13] where ≥130 steps/min also showed a higher accuracy (94%) than ≥125 steps/min (91%) and was therefore considered a superior heuristic cadence threshold. However, it is important to point out that in the two previous CADENCE-Adults studies with younger [11] and middle-aged adults [13], ≥ 130 steps/min displayed considerably higher PPV values of 70 and 49%, respectively, in comparison with the PPV of 26% demonstrated herein. These differences of magnitude are clearly driven by the total number of people capable of walking at ≥6 METs in each study. In the younger adults’ study [11], 55/76 (72%) participants, representing 64 treadmill walking bouts, achieved a vigorous intensity of ≥6 METs. In the middle-aged adults’ study [13], 33/80 (42%) participants, representing 38 treadmill walking bouts, achieved such an intensity. In contrast, only 6/98 (6%) older adult participants (representing six treadmill walking bouts) in the current study reached an intensity of ≥6 METs. By design, treadmill protocol termination decisions were implemented according to a priori determined conservative criteria that intentionally erred on the side of participant safety. Therefore, although ≥130 steps/min is a technically-correct heuristic cadence threshold for absolutely-defined vigorous intensity that is consistent across the adult lifespan that includes 21–40-year-old [11] and 41–60-year-old adults [13], the data collected herein are limited to determine a confident calibration of this threshold in our sample of adults 61–85 years of age.

Two previous studies from other research groups have analyzed the relationship between cadence and absolutely-defined intensity in samples of older adults [12, 17]. Importantly, these two studies included relatively small sample sizes (Peacock et al. [17]: *N* = 29; and O’Brien et al. [12]: *N* = 19), and limited and younger age averages (Peacock et al. [17]: mean age = 71.3 ± 6.9 years; and O’Brien et al. [12]: mean age = 68.8 ± 2.3 years). Peacock et al. [17] conducted a self-selected speed (i.e., low, medium, high) treadmill protocol only in older women, directly observed and tallied their cadence, used indirect calorimetry to assess intensity as oxygen consumption converted to METs, and utilized linear models to describe the cadence-intensity relationship. Despite the differences in testing protocols and analysis from the current study, Peacock et al. [17] also reported that a threshold of ~ 100 steps/min (99 steps/min to be precise, according to personal communication with Dr. David Rowe, a coauthor on a previous review [3]) was associated with absolutely-defined moderate intensity. In contrast, O’Brien et al. [12] reported moderate intensity-related thresholds of 104.3, 107.7, and 108.2 steps/min based on ROC analysis, a multiple regression model, and a mixed effects model, respectively, using a staged treadmill protocol consisting of five speeds ranging from 1.5–4.0 mph, a direct observation measurement of cadence and an indirect calorimetry measurement of intensity. They concluded that older adults achieved absolutely-defined moderate intensity at ~ 110 steps/min, although they did not specify the method whereby they arrived at that final approximation. The discrepancies observed between the cadence thresholds proposed by O’Brien et al. [12] may be attributable to a number of factors. The O’Brien et al. study [12] only included 19 older adults 65–74 years of age compared to the 98 older adults 61–85 years of age recruited herein. O’Brien et al. [12] conducted only 5 speed bouts ranging from 1.5–4.0 mph, whereas we employed a protocol starting at 0.5 mph and ranging incrementally up to 6.0 mph. Also, reasons for terminating the testing protocol differed; while in O’Brien et al. [12] participants were stopped if they exceeded 85% of their age predicted heart rate maximum and/or self-reported an RPE > 17, in our study participants stopped if they exceeded 75% of their age predicted heart rate maximum and/or self-reported an RPE > 13. Finally, while O’Brien et al. [12] used curvilinear methods, including multiple and mixed model regression and ROC analysis, and accounted for BMI in their proposed cadence thresholds, we used a segmented regression model and ROC analysis and a transparent evaluation process for identifying heuristic cadence thresholds based on the tradeoffs between these two analyses, and did not find any potential influence of any of the tested anthropometric variables. It is interesting to note that the moderate intensity threshold of 104.3 steps/min identified by O’Brien et al. [12] using ROC did not differ much from the ≥100.3 (95% CI: 95.7–103.1) steps/min that we identified using the same analytical approach.

A third study of older adults by Serrano et al. [24] conducted with 121 participants (68.6 ± 7.8 years of age, 65% women) explored the relationship between device-derived (i.e., not directly observed/tallied) cadence and an indicator of relatively-defined intensity. Participants were instructed to walk 200 m overground with a Garmin pedometer attached to their foot and wearing a portable metabolic cart to measure intensity. Participants were paced by a researcher walking alongside them and the test ended after maintaining, for 2 min, the cadence at which they had previously reached 40% of VO_2reserve_ (a cardiorespiratory fitness-based marker of relatively-defined moderate intensity during a maximum fitness test). Serrano et al. [24] concluded that a cadence of 115 steps/min was associated with relatively-defined moderate intensity. Discrepant findings from the current study and that conducted by Serrano et al. [24] can be explained primarily by the different definitions of intensity (absolute vs. relative [24]) applied, but also to methodological differences in protocol (paced overground vs. treadmill walking), cadence assessment (direct observation vs. a Garmin pedometer [24]), and/or the statistical methods employed (segmented regression model and ROC herein vs. linear methods [24]). Another speculative explanation may be that higher fitness levels would allow the participants in the Serrano et al. [24] study to achieve elevated cadence levels associated with relatively-defined moderate intensity thresholds or higher. To be clear, the heuristic cadence thresholds based on absolutely-defined intensity identified herein are not directly comparable to relatively-defined intensity. Although these two different intensity definitions are both based on measures of oxygen consumption from indirect calorimetry, they are neither synonymous nor interchangeable [27]. In fact, they are typically used in different applications. Descriptions of absolutely-defined intensity are more common for communicating public health recommendations of physical activity [28], whereas relatively-defined intensity is useful for developing individualized exercise prescriptions, such as in exercise programming and rehabilitation. However, we reiterate that the Serrano et al. study [24] did not use a criterion standard of direct observation to tally cadence. Future studies are needed to address this limitation when computing appropriate heuristic cadence thresholds with indices of relatively-defined intensity across the full adult lifespan.

Despite articulated concerns [3, 10, 12, 17, 24] regarding inclusion of potential individual predictors, sex, age, leg length, or BMI did not improve the predictive capabilities of the original segmented regression model that included cadence as the sole explanatory variable. In all cases, a marginal R^2^ = 0.74 was observed. Findings reported for older adults by other researchers are inconsistent in this regard and likely reflect sample-specific variations in such variables [29]. Peacock et al. [17] observed that absolutely-defined intensity was best predicted when height and age were included in the model together with cadence (R^2^ = 0.50; R^2^ = 0.25 with cadence alone in the model). On the other hand, Serrano et al. [24] reported that the association between cadence and relatively-defined intensity was best predicted when both body weight and self-selected walking cadence were included in the model (R^2^ = 0.34; R^2^ not reported for the model with cadence alone). In contrast with these two studies, O’Brien et al. [12] observed that neither height, leg length, nor body weight improved the predictive capabilities of the final cadence-intensity relationship model, while BMI did increase the ability of the model to predict cadence from absolute intensity (R^2^ = 0.77; R^2^ not reported for the model with cadence alone). As noted above, the use of a relative definition of intensity by Serrano et al. [24], hampers direct analytical comparisons with our study that used an absolute definition. Overall, the more likely explanation of the apparent disagreement across all studies may be the natural variations in sample characteristics. For instance, the study of Peacock et al. [17] performed the analyses in a sample of only women, and participants from the study of O’Brien et al. [12] showed much longer leg lengths (96.2 ± 7.6 cm) than ours (79.9 ± 5.2). Also, while the Peacock et al. [17] and O’Brien et al. [12] studies recruited participants representing relatively broad age ranges (60–87 and 65–74, respectively) they also had small sample sizes (*N* = 29 and 19, respectively). In contrast, our findings are more generalizable as a product of having the largest and most-structured (by sex and age) older sample to date.

Rather than attempting to rationalize precise cadence values which would likely not be widely generalizable for all older adults, we opted to offer evidence-based yet heuristic cadence thresholds associated with absolutely-defined intensity benchmarked by MET levels. To be clear, heuristic values are rounded, practical numbers that are intentionally grounded in evidence but are not individualized and therefore are not to be interpreted as precision estimates. Herein and in the previous installments from the CADENCE-Adults [11, 13] we identified heuristic values using a pre-determined standardized reconciliation process, considering analysis-specific values obtained from segmented regression and ROC analyses. Although there are commercially available devices that track cadence in real time [30], the heuristic thresholds proposed in the present study can be used directly by older adults themselves to easily evaluate their walking intensity in real time by simply mentally counting the steps they take in 10 or 15 s (using a watch or other timing device) and then multiplying the result by 6 or 4 (as appropriate). Steps can also be matched to a metronome [31] or music [32] to attain desired cadences. The cadence indices identified herein can be used by researchers to analyze time-stamped step data obtained from wearable technologies to determine time spent above or below these thresholds. They may also be used to design and/or evaluate walking interventions by providing clear and actionable cadence-intensity goals. Clinicians may use these as part of therapeutic exercise prescriptions. Public health guidelines could be reprised to include these cadence thresholds as a simple yet quantifiable approach to defining moderate intensity.

To the best of our knowledge, this is the first study to provide intermediary heuristic cadence-intensity thresholds of ≥4 and 5 METs in a sample of older adults, since other studies have focused only on those associated with ≥3 and/or 6 METs [12, 17]. In a preliminary 2005 study focused on younger adults, we proposed that increments of approximately 10 steps/min corresponded to an increase in intensity of 1 MET [6]. We have now confirmed this finding in the two previous installments of the CADENCE-Adults study in young and middle-age adults [11, 13] and again in the current study. Specifically, ≥ 110 and 120 steps/min have been consistently supported as heuristic cadence thresholds associated with ≥4 and 5 METs, respectively across the adult lifespan from 21–85 years of age. This claim is justified by the fact that herein we observed similar accuracy values for these specific cadence-intensity heuristic thresholds associated with classifying walking at ≥4 and 5 METs (85 and 91%, respectively) in comparison with those reported in younger and middle-aged adults from the CADENCE-Adults study (accuracy ranged from 87 to 92%) [11, 13]. As per the 2018 U.S. Federal Physical Activity Guidelines, accumulating time at an intensity of ≥4 METs (compared with 3 METs) will yield overall higher MET-minutes [21]. Since we found that relatively few older adults could actually achieve the traditionally accepted value of ≥6 METs for absolutely-defined vigorous intensity, we believe that providing these intermediate cadence-intensity heuristic thresholds is especially important for this age group given the recognized dose-response relationship between physical activity intensity/volume, function, and health [21].

No study is perfect and limitations must always be acknowledged. As alluded to above, while the heuristic cadences proposed here are rounded, practical values intended to be broadly generalizable, the trade-off is that they are limited in terms of precision, and more specifically, applicability to any single individual. It is clear that inter-individual differences exist and group-based values are shaped by the characteristics of constituent participants. Although we attempted to account for these potential inter-individual differences by including several biological and anthropometrical factors (i.e., sex, age, leg length, BMI) as additional explanatory variables, unexplained variance for cadence-intensity relationship may be due to unassessed factors, including, for example, fitness and/or habitual physical activity behavior [33, 34]. Moreover, although a cadence threshold of ≥100 steps/min appears to be a valid heuristic threshold associated with absolutely-defined moderate intensity during self-paced overground corridor walking in 21–40-year-old adults [35], it remains unknown whether these treadmill-derived heuristic cadence-intensity thresholds are similarly applicable to overground walking in older adults. The main strength of the present investigation is that this is the largest sex- and age-balanced sample of older adults used to establish heuristic cadence thresholds associated with absolutely-defined ambulatory intensity. As part of the larger CADENCE-Adult studies, the current study included a criterion measurement of directly observed and tallied cadence, indirect calorimetry to assess oxygen consumption translated to an accepted indicator (METs) of absolutely-defined physical activity intensity, and based the final set of heuristic cadence-intensity thresholds on two robust statistical analyses (i.e., segmented regression and ROC).

## Conclusion

The present investigation completes a series of reports arising from the larger CADENCE-Adults study that set out to establish heuristic cadence-based walking thresholds associated with markers of absolutely-defined intensity in ambulatory and ostensibly healthy adults across the adult lifespan of 21–85 years of age. We confirm that heuristic thresholds of ≥100, 110, and 120 steps/min correspond to absolutely-defined ambulatory intensities of ≥3, 4 and 5 METs, respectively, in 61–85-year-old adults and that these values are consistent with those identified in younger and middle-aged adults [11, 13]. Although ≥130 steps/min was technically superior as a heuristic cadence-intensity threshold associated with absolutely-defined vigorous intensity, the fact that few older adults could achieve ≥6 METs makes this specific threshold impractical and unrealistic for the majority of people in this age group. Identified heuristic cadence-intensity thresholds are useful for researchers and frontline health care providers as they can be used to establish cadence-based goals for use in walking intervention studies that pursue intensity-associated health benefits, as well as to inform the general public of cadence-based walking recommendations.

## Supplementary Information

**Additional file 1.** Table displaying final analytical data set.

**Additional file 2.** Table displaying a data dictionary for SDC 2.

**Additional file 3.** Table displaying a classification accuracy analysis for 125 steps/min as a candidate heuristic cadence threshold for 6 METs.

**Additional file 4.** Graphical representation of classification accuracy of ≥125 steps heuristic cadence thresholds and ≥ 6 METs.

## Data Availability

All data generated or analyzed during this study are included in this article and its additional files.

## Abbreviations

AUC: Area under the curve
BMI: Body mass index
METs: Metabolic equivalents
mph: Miles per hour
NPV: Negative predictive value
PI: Prediction interval
PPV: Positive predictive value
ROC: Receiver operating characteristic
RPE: Rating of perceived exertion
Se: Sensitivity
Sp: Specificity
VO2: Oxygen consumption
