# Supplementary material for: Walking cadence (steps/min) and intensity in 61–85-year-old adults: the CADENCE-Adults study
Source: Int J Behav Nutr Phys Act. 2021 Sep 23;18:129. doi: 10.1186/s12966-021-01199-4 (PMC8461976; doi:10.1186/s12966-021-01199-4)
Supplement: Supplementary file 3 — Additional file 3. Table displaying a classification accuracy analysis for 125 steps/min as a candidate heuristic cadence threshold for 6 METs. [file 12966_2021_1199_MOESM3_ESM.docx]

**Additional file 3.** Classification accuracy values of 125 steps/min as heuristic cadence threshold for vigorous intensity based on regression and ROC curve analyses.

|  |  | **Regression thresholds** | | | **ROC thresholds** | | | **Heuristic thresholds** | |
| --- | --- | --- | --- | --- | --- | --- | --- | --- | --- |
| **Intensity**  **METs** | Measure | Value | 95% PI | | Value | | 95% CI | Value | |
|  |  |  |  |  | |  | | |  |
| 6 | Threshold (steps/min) | **142.9** | 131.8 – 148.4 | **128.6** | | 128.3 – 136.4 | | | **125** |
|  | Se | 33.3 | - | 100.0 | | – | | | 100 |
|  | Sp | 99.8 | - | 97.1 | | – | | | 94.4 |
|  | PPV | 66.7 | - | 27.3 | | – | | | 16.2 |
|  | NPV | 99.3 | - | 100.0 | | – | | | 100.0 |
|  | Accuracy | 99.1 | - | 97.2 | | – | | | 94.5 |
|  | AUC | – |  | 0.99 | | 0.98 – 1.00 | | |  |
| Segmented regression and Receiver Operating Characteristic (ROC) thresholds are represented as means (95% Prediction Intervals) for segmented regression and means (99% Confidence Intervals) for ROC. Trade-offs in terms of Sensitivity (Se), Specificity (Sp), Positive Predictive Value (PPV), Negative Predictive Value (NPV) and overall accuracy between the thresholds derived from the segmented regression and ROC analyses were considered to select heuristic thresholds. Selected heuristic thresholds reflect a purposely favored tolerance for false-negative versus false-positive classifications. AUC = Area under the curve, CI = Confidence Intervals, PI = Prediction Intervals | | | | | | | | | |
