# Supplementary figures and images for: Walking cadence (steps/min) and intensity in 61–85-year-old adults: the CADENCE-Adults study
Source: Int J Behav Nutr Phys Act. 2021 Sep 23;18:129. doi: 10.1186/s12966-021-01199-4 (PMC8461976; doi:10.1186/s12966-021-01199-4)

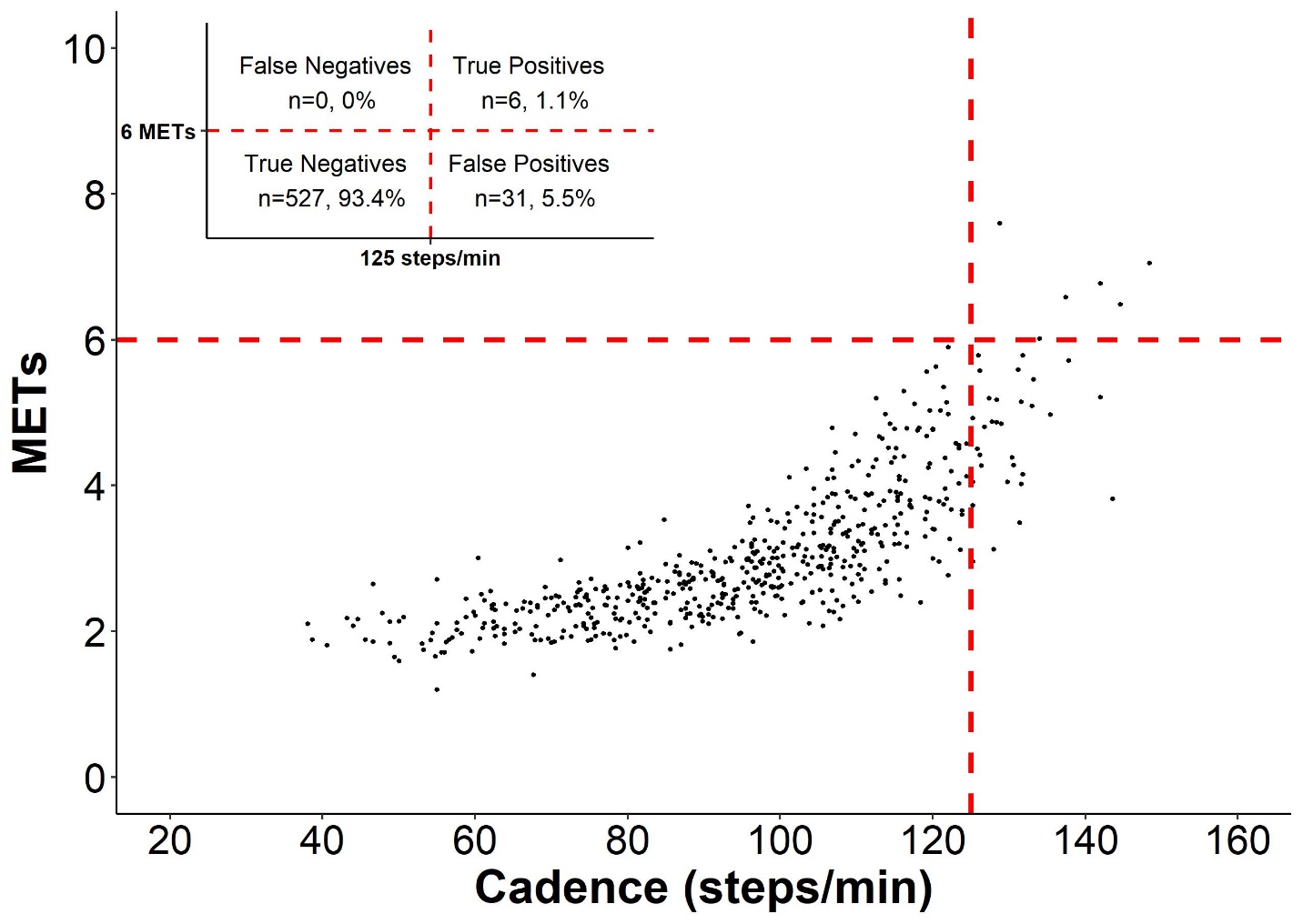


Additional file **4**. Classification accuracy of ≥ 125 steps heuristic cadence thresholds and ≥ 6 METs).

Supplement: Supplementary file 4 — Additional file 4. Graphical representation of classification accuracy of ≥125 steps heuristic cadence thresholds and ≥ 6 METs. [file 12966_2021_1199_MOESM4_ESM.docx]
